# Disrupted Saccade Control in Chronic Cerebral Injury: Upper Motor Neuron-Like Disinhibition in the Ocular Motor System

**DOI:** 10.3389/fneur.2017.00012

**Published:** 2017-01-26

**Authors:** John-Ross Rizzo, Todd E. Hudson, Andrew Abdou, Yvonne W. Lui, Janet C. Rucker, Preeti Raghavan, Michael S. Landy

**Affiliations:** ^1^Department of Rehabilitation Medicine, New York University Langone Medical Center, New York, NY, USA; ^2^Department of Neurology, New York University Langone Medical Center, New York, NY, USA; ^3^Rutgers School of Biomedical and Health Sciences, New Brunswick, NJ, USA; ^4^Department of Radiology, New York University Langone Medical Center, New York, NY, USA; ^5^Department of Psychology and Center for Neural Science, New York University, New York, NY, USA

**Keywords:** cortex, saccades, stroke, latency, disinhibition

## Abstract

Saccades rapidly direct the line of sight to targets of interest to make use of the high acuity foveal region of the retina. These fast eye movements are instrumental for scanning visual scenes, foveating targets, and, ultimately, serve to guide manual motor control, including eye–hand coordination. Cerebral injury has long been known to impair ocular motor control. Recently, it has been suggested that alterations in control may be useful as a marker for recovery. We measured eye movement control in a saccade task in subjects with chronic middle cerebral artery stroke with both cortical and substantial basal ganglia involvement and in healthy controls. Saccade latency distributions were bimodal, with an early peak at 60 ms (anticipatory saccades) and a later peak at 250 ms (regular saccades). Although the latencies corresponding to these peaks were the same in the two groups, there were clear differences in the size of the peaks. Classifying saccade latencies relative to the saccade “go signal” into anticipatory (latencies up to 80 ms), “early” (latencies between 80 and 160 ms), and “regular” types (latencies longer than 160 ms), stroke subjects displayed a disproportionate number of anticipatory saccades, whereas control subjects produced the majority of their saccades in the regular range. We suggest that this increase in the number of anticipatory saccade events may result from a disinhibition phenomenon that manifests as an impairment in the endogenous control of ocular motor events (saccades) and interleaved fixations. These preliminary findings may help shed light on the ocular motor deficits of neurodegenerative conditions, results that may be subclinical to an examiner, but clinically significant secondary to their functional implications.

## Introduction

Interventions that drive neurorehabilitation are centered on strategies to restore motor ability and improve function. However, restoration of motor ability does not ensure gains in function (1, 2). We propose that a barrier to functional progress post-injury may be the lack of understanding and characterization of subtle eye movement deficits that have been found in individuals with unilateral cerebral damage (3, 4). Impaired eye movements can impede visually guided movements, such as eye–hand coordination (5–8), which can impact function. In this study, we assess eye-movement control in a paradigm used previously to study upper limb control in chronic stroke as an initial step toward advancing knowledge of poststroke eye–hand coordination (9, 10). This may provide further insight into characterizing the ocular motor control of chronic cerebral injury in neurodegeneration.

A central element of eye–hand coordination is the timing and accuracy of eye movements that enable the acquisition of visual information (11, 12). Studies have shown that highly skilled athletes, in whom excellent eye–hand coordination is critical, utilize more efficient eye movement strategies relative to novices (8, 13–15). For example, an elite volleyball player, as compared to a novice, performs fewer fixations, of longer duration, to extract more task-relevant information, suggesting that visual strategy may coincide with skill (15). In fact, comparisons between different players in various positions engaged in the same sport reveal disparate strategies or patterns of eye control, serving their particular role on the team. For example, a defensive player uses different visual search behavior when compared with an offensive player on a soccer team (16). These results underscore the crucial role of eye movements in a dynamic environment that integrates coordinated eye and limb motion (17).

Visual dysfunction following cerebral injury can be divided into sensory (including visual acuity and visual field), motor (including extraocular muscle control), and perceptual (including neglect) disorders (18). Given this framework, previous work has verified that hemispheric stroke can significantly alter ocular motor control, including control of fast eye movements (saccades). These deficits often go undetected without objective recording techniques (3, 4, 19–22). Recent work has described the ocular motor system as a sensitive marker in ischemic stroke for motor and cognitive recovery (23, 24). The neuroanatomic underpinnings for human eye movement control, now better understood through work involving transcranial magnetic stimulation and functional imaging (19, 20), emphasize the importance of a large interconnected network of cortical and subcortical structures. The frontal eye field (FEF) and the parietal eye field (PEF) are critical control centers for intentional and reflexive saccades (25, 26). In addition, the PEF has been considered necessary for perceptual (27, 28) and value-based decision-making (29). The supplementary eye field (SEF) is considered a monitoring area to evaluate the context and consequence of eye movements, regulating saccade production during performance and for anticipated task requirements (30, 31). The pre-SEF contributes to learning motor programs while the dorsolateral prefrontal cortex (DLPFC) contributes to saccade inhibition, prediction, spatial working memory, and motor learning, along with the striatum (20, 32–34). Moreover, basal ganglia circuits have been highlighted as an intermediate step between cortical eye fields and the superior colliculus (SC) (35–40).

The existence of this large and pervasive network suggests that cerebral injury, in either the acute or more chronic stage, as in neurodegeneration, has a high likelihood of affecting ocular motor control. Given the importance of ocular motor control in eye–hand coordination and the capacity to leverage ocular motor control as a marker of recovery, a better understanding of the properties of saccades poststroke may yield insights into persistently impaired eye–hand coordination. In this study, we tested eye movement control in chronic, middle cerebral artery (MCA) stroke, relative to healthy controls, in a flashed target (intentional), saccade paradigm following a similar trajectory pattern that was used to assess limb coordination in reaching studies (9, 32, 41). We hypothesized that chronic stroke subjects without obvious visual deficits on bedside testing would show abnormal saccadic control compared to healthy controls.

## Materials and Methods

The study was approved by the Institutional Review Boards of New York University and New York University School of Medicine. Informed consent was obtained according to the Declaration of Helsinki (42, 43).

### Subjects

Twenty-six subjects participated in the study: 16 control (aged 54.8 ± 20.0) and 10 stroke subjects (aged 48.3 ± 15.1). Four of the stroke subjects had right hemispheric strokes and six had left hemispheric strokes (Table 1).

**Table 1:** **Clinical characteristics of stroke subjects**.

Subject ID	Age (years)	Sex	H/H^a^	Stroke characteristics^b^	Chronicity (years)	Fugl-Meyer score^c^
1	55	M	R/R	L middle cerebral artery (MCA) infarct: basal ganglia	3.1	60
2	45	M	R/L	R MCA infarct: corona radiata and basal ganglia	4.9	31
3	49	M	L/R	L MCA infarct/bleed: frontal, parietal, temporal lobes and basal ganglia	4.8	24
4	25	F	R/L	R MCA infarct/bleed: frontal, parietal, temporal lobes and basal ganglia	3.6	65
5	32	F	R/L	R MCA infarct: frontal, parietal lobes and basal ganglia	7.8	49
6	68	M	R/R	L MCA infarct: frontal, parietal lobes, corona radiata and basal ganglia	9.4	51
7	71	F	R/R	L MCA infarct: parietal lobe, corona radiata and basal ganglia	10.5	59
8	41	M	R/L	R MCA infarct/bleed: frontal, temporal, occipital lobes and basal ganglia	6.1	44
9	38	M	R/R	L MCA infarct: frontal, parietal, temporal lobes and basal ganglia	7.6	28
10	59	M	R/R	L MCA infarct: corona radiata, thalamus and basal ganglia	5.3	15
Avg (SD)	48.3 (15.1)				6.3 (2.4)	42.6 (17.1)

*^a^“H/H” = handedness/hemiparesis: handedness (as assessed by Edinburgh)/hemiparesis laterality*.

*^b^“Stroke characteristics”: lesion location obtained from imaging and based on detailed reports from a neuroradiologist (Yvonne W. Lui)*.

*^c^“Fugl-Meyer Score”: a summation of the Upper Extremity Score (out of 66), which reflects the extent of poststroke motor impairment*.

### Apparatus

Subjects viewed a 21” liquid crystal display monitor at a distance 42.5 cm in a dark room; the head was stabilized in a chin + forehead rest. Saccadic eye movements were monitored using a video-based EyeLink 1000 eye tracker (SR Research, ON, Canada) sampling at 500 Hz with a spatial accuracy of 0.25–0.5°*;* recordings were performed monocularly in the remote/tabletop mode.

### Inclusion/Exclusion Criteria

We recruited subjects with either right or left hemiparesis, meeting the following criteria: (1) age >21 years; (2) radiologically verified stroke in the MCA distribution >4 months; (3) ability to complete a full range of eye movements in horizontal and vertical directions, as assessed by the experimenter; (4) ability to complete the Fugl-Meyer Scale to define arm motor impairment (44–46); (5) willingness to complete all clinical assessments and experiments; and (6) ability to give informed consent and HIPPA certifications. Subjects were screened for visual abnormalities, as described below and were excluded if any obvious visual abnormalities were detected. The exclusion criteria were: (1) significant injury to the eye, weakness in extraocular muscles or to the visual system or vision in general, including the presence of visual field cuts or neglect; (2) significant cognitive dysfunction, as defined by a score <23 on Folstein’s Mini-Mental Status Examination (47); (3) clinical depression, as defined by the Geriatric Depression Scale score >11; (4) major disability, as defined by the modified Rankin Scale >4 (48); and (5) previous neurological illness or complicated medical condition precluding the completion of the experimental protocol.

Subjects were screened to ensure that there were no confounding visual deficits on the Beery-Buktenica Developmental Test of visual–motor integration (VMI), as defined by the Beery VMI (49–51), standard clinical tests for visual acuity, as defined by the Snellen chart (52), and visual field testing, assessed by confrontation testing [if in question, Goldmann or Humphrey perimetry were performed to rule out homonymous hemianopia (53)]. Hemispatial neglect was ruled out with Schenkenberg’s line bisection test (54) and the single-letter cancelation test (55). Inability to bisect a straight line within 5% of the midpoint and more than three omission errors on the letter cancelation test without evidence of field deficits on testing were taken to indicate the presence of neglect (56).

### Procedure

**Figure 1:**
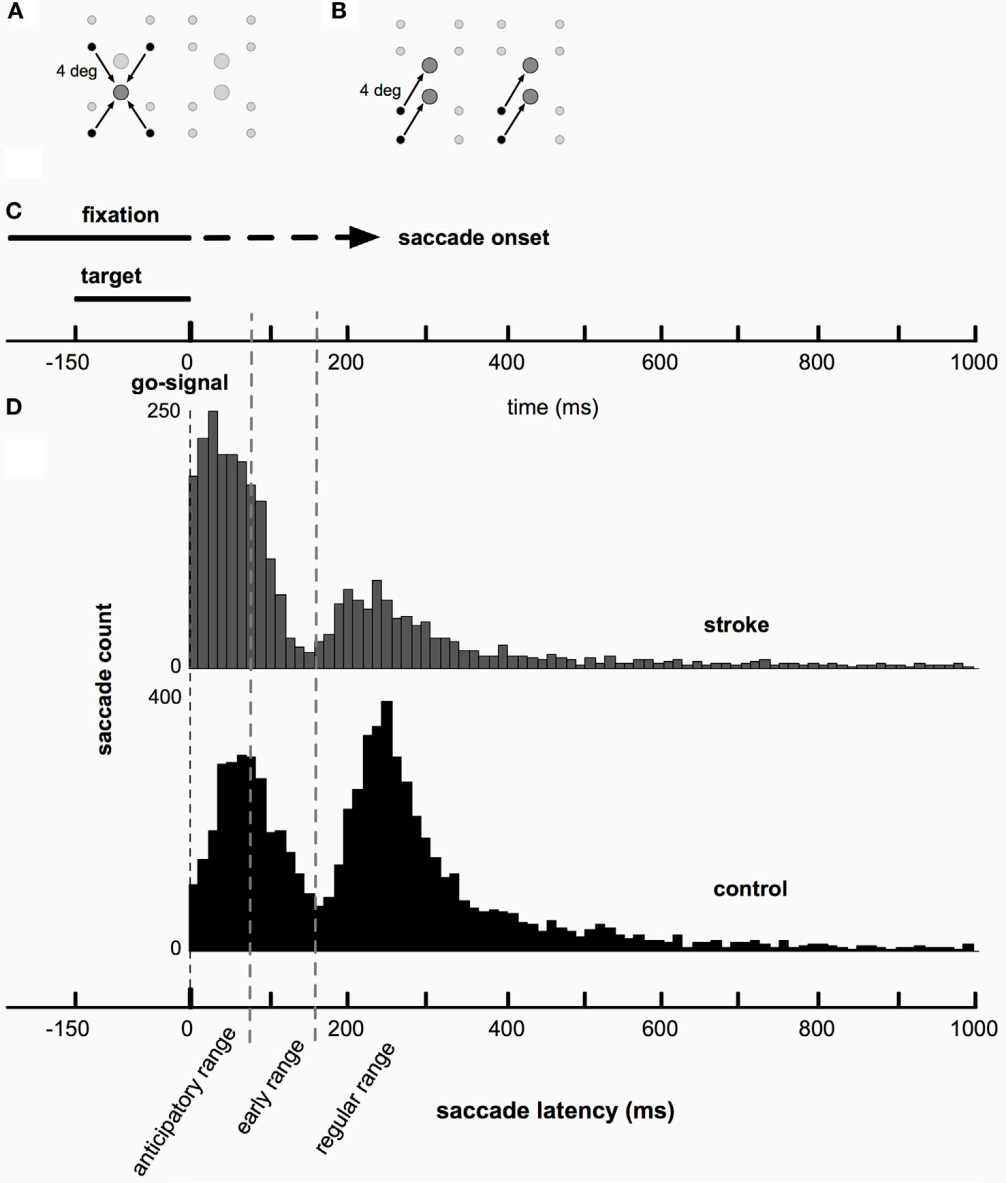
**Saccade task and timing**. **(A)** Target locations (large circles) and corresponding start locations (small circles). Emphasized with arrows: one target grouping of the target-grouped condition. The set of all stimuli (four targets and corresponding start positions) is centered on the upright computer monitor. **(B)** Target locations and corresponding start locations. Emphasized with arrows: one vector grouping for the vector-grouped condition. **(C)** Presentation of fixation and saccade targets relative to the timing of saccade onset. A fixation dot was presented at the start of each trial. While the fixation remained onscreen, one of four possible targets was presented. Saccade onset was constrained to occur only after the offset of the saccade target (150 ms following its presentation). The fixation dot remained onscreen until saccade onset was detected (dashed portion of fixation line). Early saccades were rejected and those trials repeated. **(D)** Histograms of stroke (gray) and control (black) saccade latencies. Note that there were a greater number of control subjects, who completed a greater number of saccades, than for stroke subjects. Both groups show bimodal latency distributions, with both groups displaying a large early peak at between 50 and 75 ms and a smaller secondary peak near 250 ms.

At the start of each trial, subjects were instructed to fixate a small white dot (“start position”) on a computer screen with a black background. After fixation became stable (gaze velocity had fallen below 40°/s for 1250 ms), a target dot was flashed for 150 ms (Figure 1A). Subjects were instructed to saccade to the remembered target location as soon as possible following simultaneous offset of the target and start dots (the “go” signal). Saccade onset was defined as the moment the eyes reached a velocity of 75°/s while having moved at least 0.75°, and offset was defined as the moment gaze velocity fell below 40°/s. If the eye was in motion toward the target at the time the target was extinguished, the entire screen flashed gray to indicate that the saccade had been initiated early, and the trial was repeated. All subjects were instructed to rest between trials, as needed, to prevent fatigue.

### Familiarization Saccades

Prior to making experimental saccades, subjects made 60 familiarization saccades starting from a fixation target at screen center (i.e., straight ahead) to a small target dot (0.1° radius) at a pseudo-random direction and distance. Target direction was chosen randomly and uniformly from 0° to 360°. Start-target distances were drawn from a uniform distribution (width: 1°) centered on the experimental saccade distance of 4°. That is, familiarization saccade distances were chosen randomly from the range 3.5–4.5°. Given this random selection of saccade direction and distance, familiarization targets rarely shared the same (or nearly the same) direction and distance as experimental saccades. Thus, familiarization saccades allowed us to estimate saccadic endpoint variance without providing practice with experimental saccades.

### Experimental Saccades

The design of this experiment was initially based on our previous work on reaching (9), in which reaches are patterned based primarily on the target location, or on the vector, i.e., direction and extent from start to target. Here, we present results concerning the latency, kinematics and accuracy of saccades, but the experimental design reflects that earlier work. There were four possible saccade targets arranged on a 2 × 2 grid (row spacing: 4°, column spacing: 6.5°), as shown in Figures 1A,B. Each target was associated with four possible start positions positioned 4° away from the target at directions 30°, 150°, 210°, and 330° relative to vertical. Subjects performed two blocks of saccade trials in succession. Each block consisted of nine repetitions of the 16 start-target combinations (144 per block for a total of 288 saccades per session). In one block, saccades were grouped by movement target (Figure 1A) and in the other by movement vector (Figure 1B). In the target-grouped block, all saccades corresponding to one of the four targets were performed in random order (shown for one target in Figure 1A). Then, all saccades to another target were performed, etc., until all four targets’ saccades were complete. In the vector-grouped block, all saccades defined by a particular movement vector (Figure 1B) were performed before any other movement vectors (e.g., one subject may have performed all saccades to the 30° targets, then all saccades to the 210° targets, etc., until all four vectors were completed). Note that controls were given an additional pair of target positions and two additional start positions (i.e., an additional column of two targets centered between the two columns of targets shown in Figure 1A, and an additional pair of start positions arranged horizontally to the left and right of each target, for a total six targets and six start positions around each target) as described by Hudson and Landy (9). Here, we pooled data across vector and target conditions when analyzing saccade metrics.

The visible target prior to each saccade was always a small dot (radius: 0.1°). However, the size of the to-be-acquired target (displayed after the saccade until the next start position was fixated) was determined for each subject separately at the end of the familiarization phase of the experiment. This was done systematically to equate hit rates across subjects, and was set such that it would produce an expected hit rate of 42%. As a result, the radius of the to-be-acquired target ranged from about 0.5–1° across subjects. During the experimental saccades, when the saccade endpoint was within the bounds of the target (“target hit”), the target turned blue and a reward sound was played. When the saccade did not land within the target (“missed target”), the target turned red. The proportion of “hits” was displayed continuously at the upper right of the screen.

### Calibration

Before each experimental session, subjects completed a set of center-out pursuit movements to calibrate the eye tracker to screen space. A cursor appeared at the center of the screen. Once fixated, it began to move slowly (0.8°/s) along one of the four cardinal (left, up, etc.) or four off-axis (NW, SE, etc.) directions. The cursor stopped after moving every 2.5° from the center. When fixation on the stationary cursor was stable for 1 s, the cursor moved 2.5° again until it had stopped three times (i.e., 7.5° from center). This procedure yielded 8 × 4 = 32 1-s eye-position measurements at 8 × 3 + 1 = 25 distinct screen positions from which spatial calibration was computed.

### Statistical Analysis

Raw eye-position data were initially filtered by a 3-point median filter to remove outliers. Kinematic data traces were then obtained by first aligning data to saccade onset. Average velocity traces were computed by numerically differentiating eye position within a trial, and then averaging over trials. A second numerical differentiation prior to combining data across subjects yielded acceleration traces. Peak acceleration/deceleration and velocity were defined as the corresponding peaks of the average acceleration and velocity traces.

Analysis of temporal data (saccade latency and duration) was performed on the reciprocal of latency (in units of s^−1^), which reduces the skew typically seen in temporal measurements, yielding more normally distributed data (57). Means and 95% confidence regions were computed on inverse-transformed data, yielding computed means that were close to the median of observed latencies and asymmetric confidence bounds when plotted in time units (s).

Two-sample *t*-tests were used to determine whether pairs of means or variances differed. Our results were unchanged if comparisons were made using Welch’s *t*-test, which makes use of equations designed to account for possible heteroscedasticity and unequal sample sizes (the Welch-Satterthwaite equation for degrees of freedom). As a complement to traditional *t*-tests, we have plotted Bayesian 95% confidence regions around all computed estimates in the figures; as can be seen graphically in the corresponding figures by comparing confidence bounds, Bayesian analogues of the reported *t*-tests confirm our statistical analyses. Single proportions were compared *via* the *z*-test for equality of proportions (*S*_1_ of *N*_1_ vs. *S*_2_ of *N*_2_), where *z* is:

$$\text{z}=\frac{{\text{S}}_{1}/{\text{N}}_{1}-{\text{S}}_{2}/{\text{N}}_{2}}{\sqrt{\left[\frac{{\text{S}}_{1}+{\text{S}}_{2}}{{\text{N}}_{1}+{\text{N}}_{2}}\right]\left(1-\left[\frac{{\text{S}}_{1}+{\text{S}}_{2}}{{\text{N}}_{1}+{\text{N}}_{2}}\right]\right)\left(\frac{1}{{\text{N}}_{1}}+\frac{1}{{\text{N}}_{2}}\right)}}.$$

Patterns in the number of saccade latencies occurring within each sub-stratification (see below) were compared via *χ^2^* test.

## Results

### Saccade Timing

**Figure 2:**
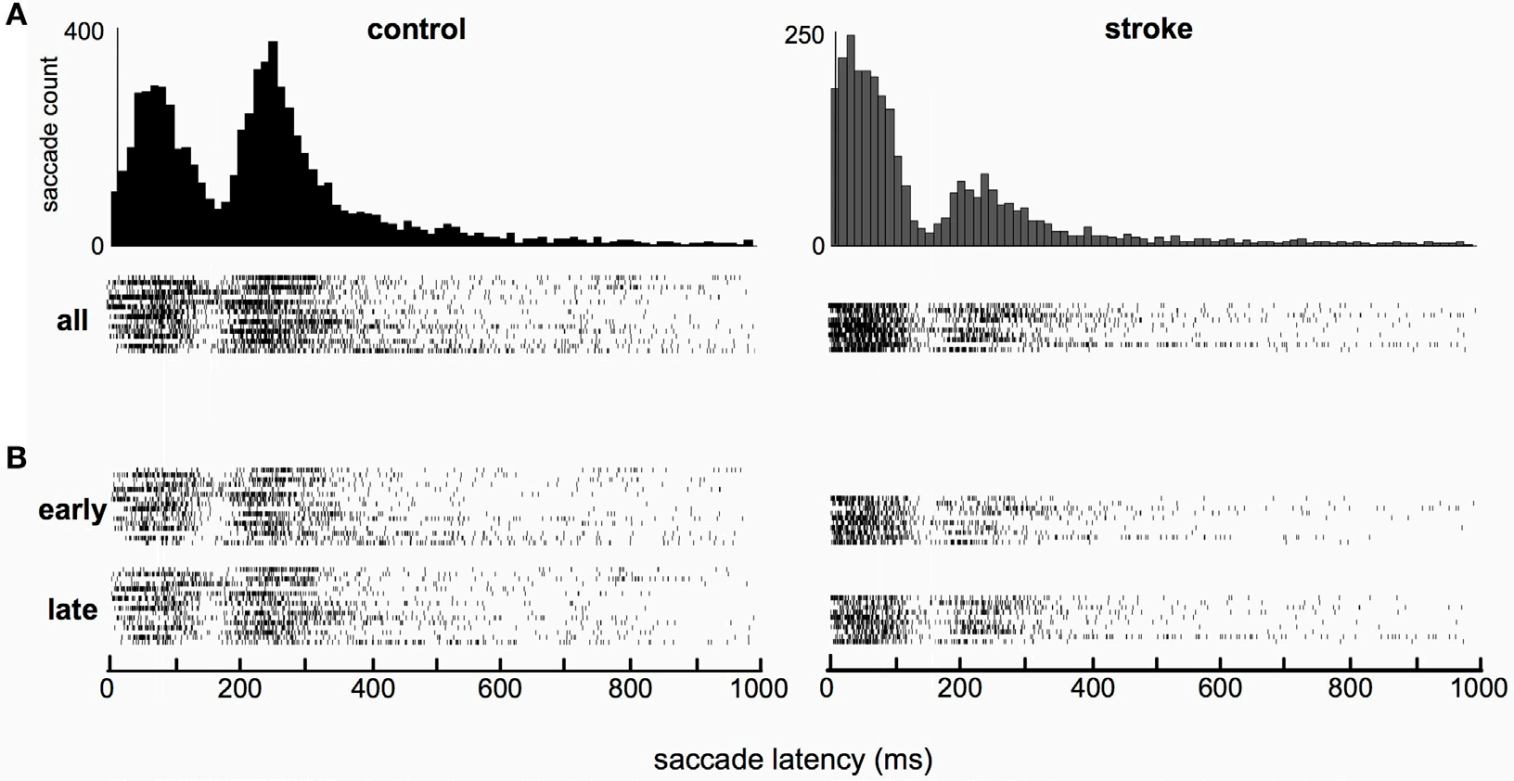
**Raster plots of individual subjects’ saccade latencies**. **(A)** All saccades. Each raster shows all saccade latencies for a single subject (16 control and 10 stroke subjects). **(B)** Saccades separated by those that occurred during the first and second halves of the session (“early” and “late”). Histograms are repeated from Figure 1 to allow easy comparison of the high-density regions of histograms and raster plots.

Distributions of saccade latencies (relative to the “go signal”) were bimodal in both groups. We separated mode one (first peak) into saccades in the anticipatory range, as defined by latencies up to 80 ms, and in the “early” range, as defined by latencies between 80 and 160 ms. Mode two (second peak) included saccades in the “regular” range with latencies above 160 ms. The average timing of saccades was significantly different in stroke subjects compared to healthy control subjects. Figure 1C shows a schematic of the timing of the task, and Figure 1D displays histograms of saccade latencies. Inspection of these histograms suggests very similar latencies for the two modes in the distributions, but that the difference in the frequency distribution of saccade latency between stroke and control subjects was due to the higher number of saccades occurring in the first mode in stroke subjects and higher number of saccades in the second mode in control subjects. This pattern occurred more or less uniformly across individual subjects (raster plots, Figure 2A) and throughout the session (Figure 2B). The distribution of latencies from the first half of each subject’s dataset was essentially identical to the pattern observed in the second half of the experiment.

**Figure 3:**
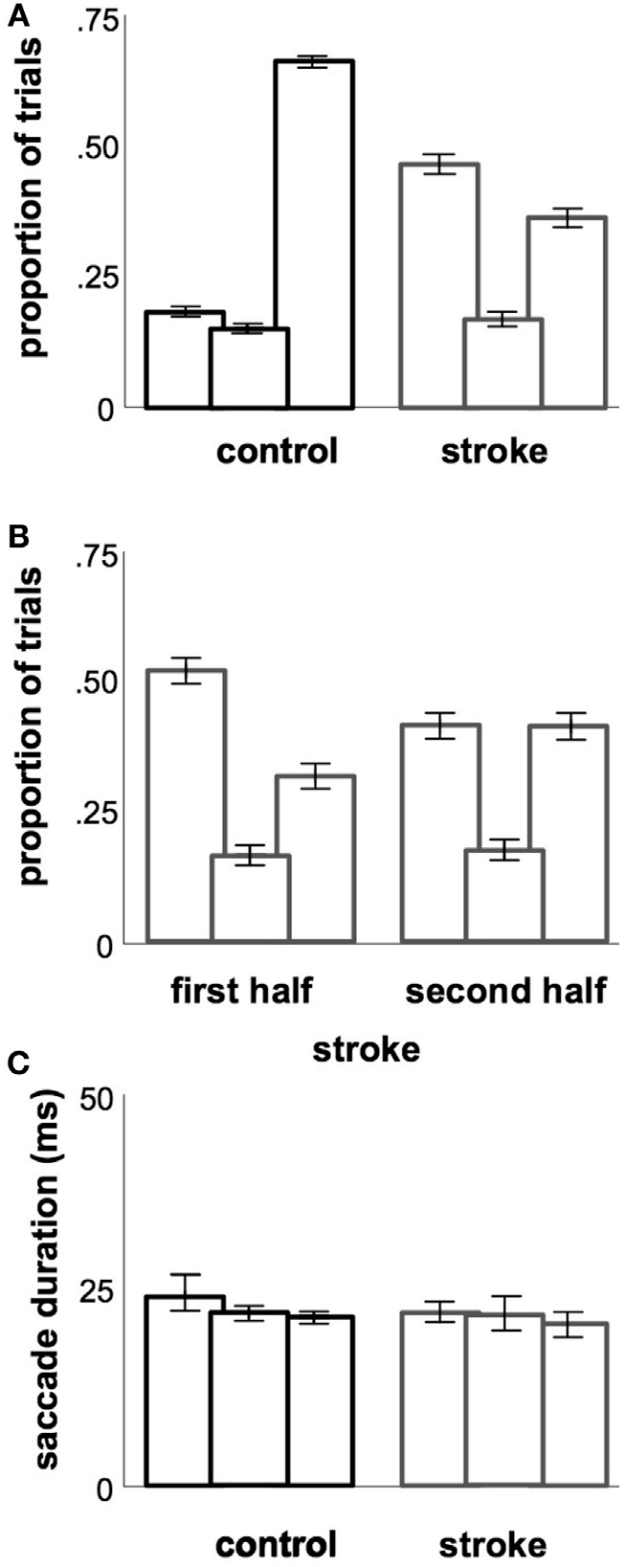
**Temporal metrics**. **(A)** Proportions of saccades occurring in each of the three latency ranges (each triple is ordered, from left to right: anticipatory, early, regular). **(B)** Proportions of saccades occurring in each latency range, as in **(A)**, but split between the first and second halves of each stroke subject’s session. **(C)** Average saccade durations occurring in each of the three latency ranges. *Error bars* (included in all plots): 95% confidence range for the mean across subjects.

Within each of the three latency ranges, we see that the overall difference in saccade latency was driven primarily by the number of saccades that fall into each of the three categories in stroke vs. control subjects. Stroke subjects displayed a disproportionate number of saccades in the anticipatory range of latencies compared to controls, whereas control subjects produced more of their saccades in the regular range compared to stroke subjects (Figure 3A, *χ^2^* = 895, *p* < 0.05). There was no difference in the pattern of saccade latencies observed in the first and second halves of the experiment in our stroke cohort (Figure 3B, *χ^2^* = 1.26, *p* > 0.05) or in controls (*χ^2^* = 2.35, *p* > 0.05). Within each of the three ranges, there were no significant differences in saccade duration between the stroke and control cohorts (Figure 3C). Note that the larger number of anticipatory saccades produced by stroke subjects also resulted in a greater proportion of rejected trials due to saccades initiated prior to the “go” stimulus in stroke subjects (22.3% of all attempted saccades) vs. controls (13.8%; *z* = 11.4, *p* < 0.05).

### Accuracy and Precision of Saccades

**Figure 4:**
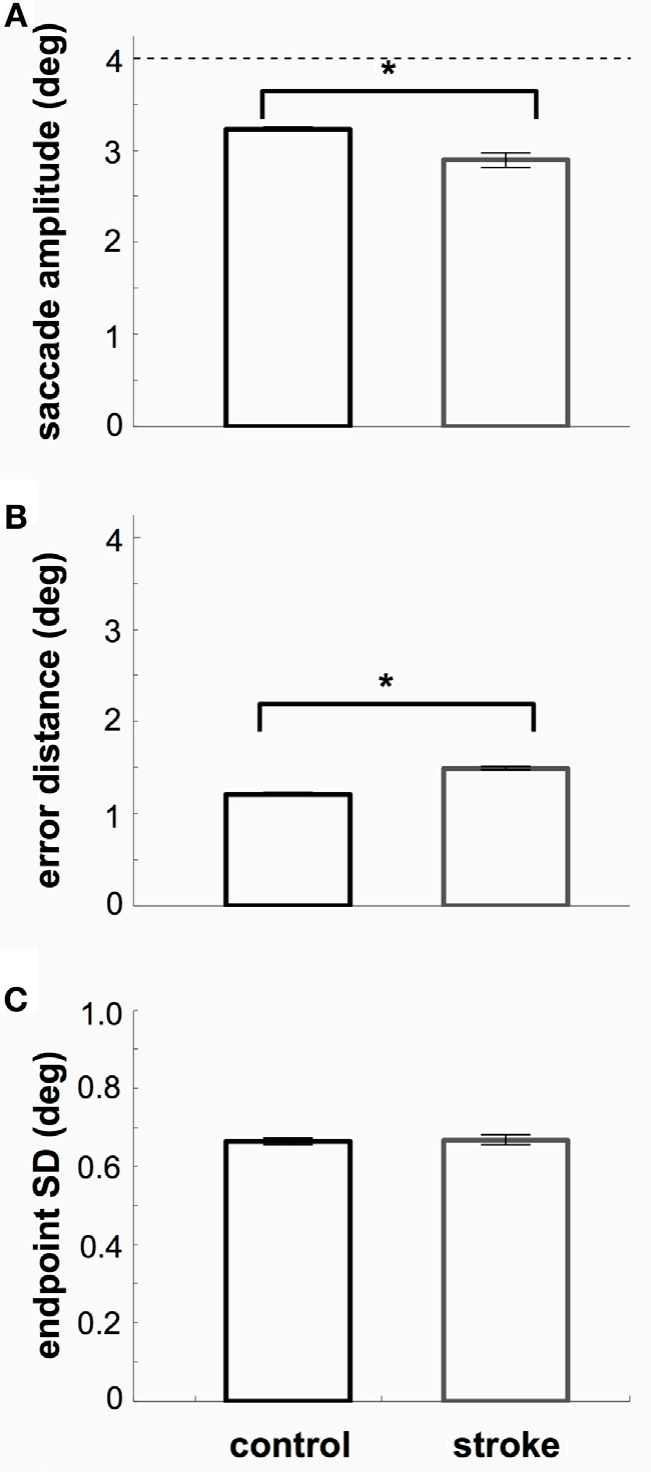
**Spatial metrics**. **(A)** Average saccade amplitude. The dashed line indicates the distance of the target. Stroke subjects are more hypometric than controls. **(B)** Average 2D distance between saccade endpoint and target. Stroke subjects are less accurate than controls. **(C)** Endpoint SD. SD was computed assuming a symmetric error distribution. Stroke subjects show less precision of saccade endpoints (relative to their mean). *Error bars* (included in all plots): 95% confidence range for the mean across subjects.

We separated saccade accuracy into two categories: the length of the saccade (saccade amplitude; Figure 4A) and the 2D distance between saccade endpoints and target center (Figure 4B). SDs are shown in Figure 4C. As expected, stroke subjects produced saccades that were more hypometric than those of controls (*t*_24_ = 7.7, *p* < 0.05), were further from the target (*t*_24_ = 20.5, *p* < 0.05), and were more variable (*t*_24_ = 7.2). Separating these measures based on whether saccades latencies were in the anticipatory, early, or regular ranges, we find that saccade amplitudes of controls increase for higher saccade latencies [2.9–3.3*°, F*(2,45) = 122.4, *p* < 0.05], and a small decrease in error magnitudes for higher saccade latencies [1.5–1.2*°, F*(2,45) = 269.2, *p* < 0.05]. Finally, there was a small increase in error distance with increasing latency for stroke subjects [1.3–1.8*°, F*(2,27) = 188.2, *p* < 0.05]. There were no other latency-dependent effects in stroke subjects or for standard errors in either subject group (all *p* > 0.05).

### Saccade Kinematics

**Figure 5:**
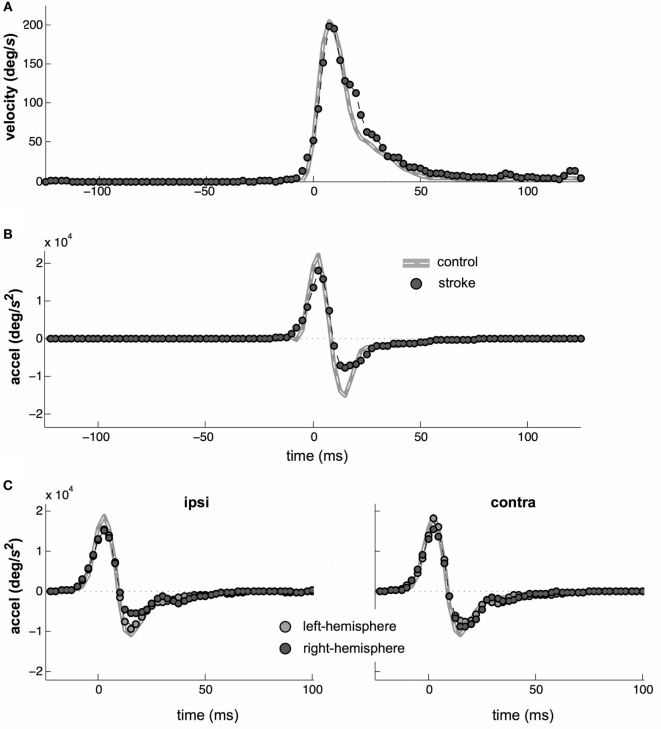
**Saccade kinematics**. **(A)** Average saccade velocities of control (gray line) and stroke (circles) subjects. **(B)** Average saccade acceleration. **(C)** Average acceleration profiles separated by left- vs. right-hemisphere stroke (light- vs. dark-gray datapoints, respectively) and by whether a particular saccade was directed toward or away from the affected field (left vs. right plots, respectively). Note that all target distances were 4°, so that main sequence effects are small.

Saccade velocity and acceleration profiles are typically highly stereotyped and saccade velocity profiles displayed the characteristic right-skewed shape for both groups (Figure 5A). However, the right-hand tail was slightly more prominent in the stroke group. This is consistent with a weaker and more prolonged deceleration phase in the velocity profile following stroke (Figure 5B). There was also a significant difference in acceleration profiles at the time of peak deceleration between control and stroke subjects (*t*_24_ = 3.4, *p* < 0.05).

### Separation by Stroke Hemisphere or Saccade Direction

Eye movement control is lateralized and saccadic deficits may be greater for saccades made into the contralesional visual field (58, 59). Therefore, we repeated all of the above analyses separately for contralesional and ipsilesional saccades. The results were nearly identical between the contralesional and ipsilesional saccade directions. In particular, the pattern of onset latencies did not vary with saccade direction. The only significant difference occurred in the saccade kinematics, where the amplitude of the deceleration phase of the saccade waveform was asymmetrically attenuated in stroke. Although sub-stratification of this result reduced the statistical power of further testing, it appeared to be primarily the result of ipsilesional saccades in right-hemisphere stroke subjects (Figure 5C), as this was the subgroup in which the peak deceleration was lowest (*t*_24_ = 1.94, *p* = 0.064) relative to the peak deceleration in control saccades.

## Discussion

We have demonstrated a variety of deficits in the control of saccades after stroke in individuals with otherwise intact visual function. Most striking among these was the disproportionate number of saccades made by stroke subjects with timing in the anticipatory range (80 ms or less). Saccades in stroke subjects were also less precise (increased variance) and less accurate, compared to healthy controls. We discuss each of these deficits in turn, paying particular attention to the possibility that they may have implications for visually guided reaching and for neurodegeneration.

### Saccade Latency Abnormalities: A Disinhibition Phenomenon

The shortest possible biological saccadic latency, reflecting transmission of information between retina and brainstem saccade generators and brainstem saccade generators to extraocular muscles for eye movement, is about 60 ms (60). However, the latency of typical saccades to unexpected peripheral targets is about 200 ms (32). This difference reflects decision-making and cognitive processing. In an experimental setting, saccades will often anticipate the relevant go-signal, resulting in latencies near or below the 60 ms limit. Here, we binned saccades with latencies less than or equal to 80 ms separately, and labeled them as within the “anticipatory” (61–63) range.

Although subjects were disincentivized to anticipate the go-signal based on task instructions and feedback (saccades made too early were rejected and repeated with a screen flash), such saccades were made by both groups in our task. Anticipation for the go-signal was possible because targets were shown prior to the go-signal and there was fixed timing of target onset and of go-signal. Nevertheless, the majority of saccades made by control subjects were in the regular range. In stark contrast, the majority of saccades made by stroke subjects were in the anticipatory range, perhaps suggesting an inability to suppress such saccades, rather than a purposeful decision to ignore instructions.

The inability to suppress saccades until the go signal (simultaneous offset of target and fixation cue) could represent a range of possible deficits, where at one end saccades occur reflexively in response to the target and, at the other, subjects inhibit saccades perfectly until instructed. In cerebral injury, the ability to maintain suppression or the ability to time the termination of saccade suppression may be impaired. All of these scenarios would create more saccades in the anticipatory range, as we see in our data, and should be considered inappropriate pro-saccade responses to the target. The most severe form, the complete inability to suppress a saccade to the flashed target, is not unlike the occurrence of what would be seen as inappropriate prosaccades during an anti-saccade task.

While neural control of saccades is distributed throughout a large network of cortical, subcortical, and brainstem structures (20, 32, 35–37, 39, 40, 64), the FEF, the PEF and basal ganglia play a role in intentional saccades (as in our flashed target task, as properly executed, suppressing an eye movement until the go signal). The last structure in this chain, at the convergence of the basal ganglia’s multiple pathways, is the substantia nigra, which is known to have an inhibitory effect on the SC (39, 40). Studies on stroke have focused on cortical lesions affecting the ocular motor network, particularly as these neurologic insults relate directly to cortical eye fields, which exert a direct excitatory effect on the SC (3, 4, 65).

Fixation neurons in the rostral pole of the SC play a critical role in the maintenance of fixation (66), and depression of activity within these neurons releases fixation (67). Fixation-related neurons have also been identified in the substantia nigra pars reticulata (68), posterior parietal cortex (69, 70), and frontal lobes (71, 72). While it is not possible to determine the net effect on the SC in our subjects, it is possible that involvement of cortical eye fields and/or substantial basal ganglia involvement in our cohort (Table 1) played a role in the observed saccadic disinhibition via alteration in tonic input to the SC. This upper motor neuron-like disinhibition in ocular motor control that may be characterized here could prove beneficial in understanding the phenomenology of both acute and chronic cerebral injury, including neurodegeneration.

### Speed-Accuracy Trade-off in Eye Movement Control

We observed a significant decrease in saccade amplitude (reflecting reduced saccade accuracy) and an increase in saccadic endpoint variability (reflecting reduced precision in the stroke group relative to controls). A well-known feature of motor behavior is the speed-accuracy trade-off (73). The saccadic main sequence (duration and peak velocity as a function of saccade amplitude) describes a relationship in which larger-amplitude saccades are more rapid and have longer duration. A feature of larger amplitude/faster saccades is poorer spatial accuracy; this represents the optimal trade-off in the face of signal-dependent noise inherent in ocular motor command signals (74). We found that accuracy and precision were both negatively affected in stroke. Rather than producing a consistent shift along the main sequence (i.e., toward lower peak velocities and lower amplitudes), these subjects show reduced saccadic amplitudes without a corresponding reduction in peak velocity as would be predicted by the main sequence relationship (75). However, to look at this deviation from the main sequence more closely would require a future study using a wider range of saccade magnitudes.

### Implications for Rehabilitation Strategies

The difference between gross motor ability and functional motor control is a key distinction that must be made when evaluating recovery from any brain injury, including stroke, of both an acute and chronic nature, i.e., neurodegeneration. The difference between these two aspects of recovery is not in whether one can move a particular effector, but in the character of that control. In stroke subjects with residual hemiparesis, we have shown that eye movement latencies in a flashed target saccade paradigm are significantly altered in the temporal domain. These deficits in saccadic control may also affect the coupling of eye and hand movements, thereby altering functional use of the arm in individuals with stroke. After all, the integration of these systems is characterized by temporal relationships (76, 77) and small shifts in eye movement timing relative to hand-movement timing (that would typically go unnoticed during standard clinical evaluation) may alter the framework on which integrated movement plans are built (78). A clearer understanding of the synchronous and interdependent control systems directing eye and limb movements will likely be key to restoring functional ability poststroke. Furthermore, recent studies have demonstrated that eye movement execution for visually guided reaches occurs simultaneously with motor planning for arm/hand movement (79, 80). When reconciled with limb motor planning deficits in chronic stroke (81), this may create computational delays and could help explain recovery plateaus or impeded rehabilitation progress. Given that eye control precedes arm control (17, 76, 77, 82), our results highlighting dysfunctional ocular motor control may prove influential in better understanding visually guided, manual motor control. The development of strategies to rehabilitate eye movement control and ultimately to improve eye–hand coordination may be critical to the restoration of function poststroke. These ocular motor findings may also set a foundation for improved understanding in eye movement control for chronic neurodegeneration.

## Author Contributions

Conception and design of the study and substantial manuscript drafting: J-RR, TH, AA, PR, JR, and ML. Acquisition and analysis of data: J-RR, TH, AA, YL, JR, and ML.

## Conflict of Interest Statement

The authors declare that the research was conducted in the absence of any commercial or financial relationships that could be construed as a potential conflict of interest.
